# Practical Notes and Formulæ

**Published:** 1878-04-20

**Authors:** 


### *PRACTICAL NOTES AND FORMULÆ.*

### LACERATION OF THE PERINEUM.

Dr. F. M. Rushing writes: "I am not aware of the following plan of procedure in laceration of the perineum having been adopted by the profession.

I will give you a complete history of the case so that you may understand the advantage claimed by the plan adopted.

Mrs. R., aged about 30, delivered of her fourth child (a boy) on the 10th of September, last—sent for a physician, but did not obtain one. The child was born without any assistance whatever. On the 14th, she discovered that the feces could not be retained. I was called in to determine the cause. Upon examination I found that the whole perineum had been torn through and that the rectum had been slit up for nearly or quite an inch. I also decided that a portion of the laceration was caused at some previous labour. I advised them to wait until the discharges stopped, and I would operate, (her health being very bad) her mother insisted that I would make an effort then. I had no instruments with me, except a surgeon's needle and some silk. I therefore made three stiches, bound the thighs together and advised milk diet, etc., and told them I had no idea it would affect anything. I heard no more from the case until the 10th of October. Her husband told me he wanted me to operate. I gave him some purgative medicine to prepare the system and told him to give it that day and night until he thoroughly cleansed out the bowels. On the 11th of October I obtained the assistance of Dr. J. G. Moore, and we operated in the usual manner. The bowels were kept bound up for 12 days and she confined strictly to milk diet. We removed the stitches at this time and found that union had not taken place for more than ½ of an inch on the vaginal or upper front portion. We did nothing more until the 14th of November, when we again prepared the system with purgatives and on the 15th we proceeded to operate the second time. Both edges of the laceration being closed, we found it very difficult to pare the surfaces. I suggested using a stick of caustic potassea; (with some doubts of its propriety my assistant consented) we accordingly made a solution of strong vinegar, half water and inserted a sponge well filled with it, into the rectum to prevent the caustic from affecting it. We then applied the caustic to the portion we wished to make raw, and immediately afterwards washed the surface thorougly with the solution of vinegar, to prevent the pot. from causing a deep destruction of the tissues. We then, with a small knife scraped and pared off the dead flesh; washing with the solution of vinegar all the time. After thoroughly cleansing the surface, stopping hemorrhage, etc., we applied three stitches as before, as is customary in such operations. The union was complete in 12 days. I will state that we confined the patient to milk diet, and kept the bowels bound up with a dose of morphine occasionally.

We think that the difficulty of obtaining a raw surface with the knife is overcome with the potash, and that with it you get a uniform raw surface on every portion desired.

[The result of this experiment affords a most valuable suggestion.—Ed.]

### BURNS.

G. W. Smith, M.D., of Arkansas, writes:

*Editors Medical Record*:

I send you treatment of burns, which I have found very efficacious in a member of my own family. My infant fell into the fire on March 3d, and burned the left hand severely. I saw her a few moments after the burn. My wife was applying coal oil in a stream upon the hand. I immediately made a thick paste of linseed oil and lime water. I took half pint linseed oil and added lime water, stirring all the while until it became of thick consistency, enveloped the hand in this and cotton to the exclusion of the air. I let it remain two days in this condition, then undressed it with same application excepting the lint cotton, which was substituted by linen cloths somewhat charred by the fire. I let it remain two days longer without dressing. At the expiration of four days I removed the dressing again, and the entire cuticle came off with it. What now was I to do to stimulate and heal? As linseed oil had done so much in relieving the little sufferer, I concluded to continue it, with addition of carbolic acid, leaving off the lime water, as follows:

R.—Linseed oil........................ oz. viii.
Carbolic ascid ........................oz i.

To be applied on every part of the burnt surface *ter die.* This application was continued one week, then substituted by the following:

R.—Ol. olivæ ......................... oz. viii.

Carbolic acid..........................oz i.

Apply to every part of burnt surface two times per day. This completed the cure. I would advise those whose duty it becomes to treat burns, to use as little water as possible to the denuded surface. Carbolic acid we find to be one of the most soothing stimulants to denuded burnt surfaces known. In this case, when the surface became dry and hot, I immediately applied the carbolized oil, which soothed and quieted, and refreshing sleep was induced. No deformity remaining.

---

### PRURITUS VULVÆ.

This affection is exceedingly annoying to the patient, and scarcely less so to the physician, because of its great obstinacy.

In treating the disease the practitioner should first seek for the cause. If this is detected, there is good hope of success in the treatment. Both constitutional and local remedies are required. It may depend upon uterine disease, or be associated with dyspepsia, constipation, or other intestinal derangement, causes which must be met by appropriate internal treatment. Locally the formulæ used have been numerous. Duhring, in his late work on skin affections, mentions most favorably camphor, chloral and borax, variously combined.

R.—Chloral ........................grs. x to xxx.
Water..................................oz. i.

Used as a lotion to the parts.

R.—Boracis.............................dr. iv.
Morphiæ sulphatis...................gr. viij.
Glycerine..............................oz. ss.
Aquæ.............................. oz. vij ss.

M. These preparations, a little weakened, may be used by injection. For this purpose the following has been found highly efficacious:

R.—Nitrate of alumina...................dr. i.
Aquæ...................................oz. x.
M.

As an ointment, the following is strongly recommended:

R.—Camphoræ,
Chloralis hydratis..................aa. dr. i.
Ungt. aquæ rosæ......................oz. i.
M.

---

### SECONDARY SYPHILIS.

A good formula for the administration of mercury in syphilis is the following:

R.—Corrosive sublimate ..................grs ij.
Comp. tinct. gentian...................oz v.
M.—Dose, one teaspoonful after each meal.

Another—
R.—Prot. iodide mercury,
pulv. opium.......................a a grs x.
Ext. gentian ............................q s.

M. Divide into pills No. 40. One night and morning.

These are well adapted to cases which are of long standing, or verging upon the tertiary stage. In tertiary syphilis, wherein mercury has been previously used, the following is a good prescription:

R.—Iodide potass. .......................oz ss.
Comp. tinct gentian.................. oz vj.

M. Dose, one to two teaspoonfuls three times a day.

### ARSENIC IN SKIN DISEASES.

Arsenic has been much relied upon as a constitutional remedy in chronic skin diseases. A good formula for using it is that recommended by Prof. Duhring:

R.—Fowler's solution of arsenic........ dr. jss.
Wine of iron q. s. to make.......... oz. iv.

Of this a teaspoonful may be given immediately after meals. The remedy requires to be persevered in for weeks, and sometimes for months, in obstinate cases. Yet it must be given with caution, and suspended for a time or dose diminished if its constitutional effects appear. Some persons cannot bear the above dose; even a single drop of Fowler's solution will not be borne by some constitutions.

### GUIACUM IN QUINSY.

Guiacum has, of late, been recommended in quinsy and other throat affections. The following formula for its use is suggested by a writer in the *St. Louis Med. News*:

R.—Potass. chlor........................ dr. j.

Spts. æth. nit........................ dr. iv.

Tinc. guiac........................ dr. iv.

Syr. aurant. cort.................... dr. vj.

M. Dose, a teaspoonful every two hours mixed in a little water. If the bowels are too freely acted upon, diminish the dose.

### HEMORRHOIDS.

R.—Pulv. rhei......................... grs. xl.
Ipecac.............................. grs. xii.
Saponis............................ grs. vj.

Divide in pills No. 15. Used by Prof. Bedford as a laxative in piles. Dose—one pill two or three times a day.

### YERBA REUMA.

The yerba reuma is a plant, herbaceous, growing near the foothills of the coast-range mountains. It passed out of flower before my attention was called to it, but in due time its name will botanically be obtained. Its Spanish name implies, flowing or flux herb. It contains largely chloride of sodium, and a peculiar astringent. It is only as a local remedy that I have ever tested it. My first test was that upon my friend, Dr. Thomas Porter, who had suffered two years from nasal catarrh. I prepared a tincture, using four ounces of the drug to a pint of alcohol (250°), and gave

R.—Tincture yerba reuma, one fluid ounce.
Aqua, three fluid ounces.

M. S. Snuff one teaspoonful from the hand through each nostril three times daily. In three weeks Dr. Porter was cured and remains so. He had tried everything recommended with but little benefit. He suggested its use in leucorrhoea. I tried it and the result in every instance was a cure. I gave it in the form of an injection in gonorrhoea, and the result was the same, one four ounce mixture performing the cure.—*New Prep.*

### TANNIN AS A MEDICAL AGENT.

There is no astringent in the materia medica so useful and so extensive in its application to the treatment of various diseased conditions as tannin. We annex a few formulas which will be found convenient and efficient for the purposes named:

#### IN MENORRHAGIA.

R.—Tannin ........................ grs. xx.
Opium .......................... grs. iv.
Ipecac ......................... grs. v.
M. Divide in powders 10.

Take one every two to three hours. The same is useful in the hemorrhage of abortion, and other forms of hemorrhage.

#### NIGHT SWEATS.

R.—Tannin........................ grs. ij.

Opium ......................... gr. i.

M. One pill to be taken at bed-time. Another excellent remedy for night sweats, uterine hemorrhage, or

#### COLLIQUATIVE DIARRHŒA.

R.—Tannin ........................ grs. xx.
Pulv. opii........................ grs. v.
Pulv. ergot....................... grs. xv.
Loaf sugar........................ grs. xxx.

Triturate and divide into ten powders. Give one every two to four hours.

